# Preference for Leaders with Masculine Voices Holds in the Case of Feminine Leadership Roles

**DOI:** 10.1371/journal.pone.0051216

**Published:** 2012-12-12

**Authors:** Rindy C. Anderson, Casey A. Klofstad

**Affiliations:** 1 Department of Biology, Duke University, Durham, North Carolina, United States of America; 2 Department of Political Science, University of Miami, Coral Gables, Florida, United States of America; Institut Pluridisciplinaire Hubert Curien, France

## Abstract

Human voice pitch research has focused on perceptions of attractiveness, strength, and social dominance. Here we examine the influence of pitch on selection of leaders, and whether this influence varies by leadership role. Male and female leaders with lower-pitched (i.e., masculine) voices are generally preferred by both men and women. We asked whether this preference shifts to favor higher-pitch (i.e., feminine) voices within the specific context of leadership positions that are typically held by women (i.e., feminine leadership roles). In hypothetical elections for two such positions, men and women listened to pairs of male and female voices that differed only in pitch, and were asked which of each pair they would vote for. As in previous studies, men and women preferred female candidates with masculine voices. Likewise, men preferred men with masculine voices. Women, however, did not discriminate between male voices. Overall, contrary to research showing that perceptions of voice pitch can be influenced by social context, these results suggest that the influence of voice pitch on perceptions of leadership capacity is largely consistent across different domains of leadership.

## Introduction

Like non-human animals [1], humans respond to non-linguistic information encoded in vocal signals. Human voice pitch–“highness” or “lowness” in fundamental frequency as determined by the size of the larynx and length and mass of the vocal folds–is sexually dimorphic (on average twice as high in women compared to men [2]), and influences how speakers are perceived. Men with lower-pitched (i.e. masculine) voices are perceived as more attractive [3], physically stronger [4], and socially dominant [5]. Women with masculine voices are also perceived to be socially dominant [6]. In contrast, however, women with higher-pitched (i.e. feminine) voices are perceived as more attractive (e.g. Jones et al. 2008).

Recent research shows that voice pitch influences the selection of leaders. Both men and women prefer male leaders with lower-pitched voices, and associate lower-pitch with traits such as integrity, strength, and competence [7], [8]. Men and women also prefer female leaders with lower-pitched voices, and similarly associate them with traits such as competence and trustworthiness [7]. In asking listeners about their perceptions of voice pitch, however, these studies did not make reference to a specific leadership position. In light of work showing that perception of voice pitch can be influenced by social context [9], the question remains whether the preference for leaders with lower-pitched voices is consistent across different leadership positions.

Research on gender roles shows that leadership is generally seen as a masculine role. Men are overrepresented in leadership roles and are perceived to be more assertive, controlling, and confident than women [10]. This perception can be moderated, however, if the leadership role in question is perceived to be feminine; that is, a position typically occupied by women, or that is congruent with the stereotype of women as caretakers of families and children [10]. Here we test the prediction that men and women will prefer male and female leaders with more feminine voices for feminine leadership roles.

We asked male and female listeners’ their preferences for male and female leaders with higher- and lower-pitched voices running in hypothetical elections for two feminine leadership roles: a Member of the School Board (a municipal-level governing body that oversees schools), and President of the Parent Teachers Organization, or “PTO” (a school-level voluntary service organization). In line with previous work on gender roles and leadership [10], both positions are concerned with the welfare of children. Also, in the United States, where our study was conducted, women are overrepresented in both positions. For example, in the largest school system in the United States, New York, NY, each of the 32 school boards in the city are comprised of more than 50% women, and 59% are led by women. Likewise, data collected by the United States Department of Education indicate that 77% of PTO participants are women (2007 Parent and Family Involvement in Education Survey).

## Materials and Methods

### Stimuli

Ten women (mean age = 28 years) and ten men (mean age = 33 years) were recorded speaking the phrase “I urge you to vote for me this November,” an electorally-relevant, yet politically neutral utterance. Recordings were made in an anechoic chamber. Each digitized audio file was inspected aurally and visually (via spectrograms using Syrinx, www.Syrinx-PC.com version 2.6 h), to ensure that utterances were free from speech errors and non-speech noise. The amplitude of each utterance was normalized using the Signal acoustic analysis program (Engineering Design version 4.02.04).

We used the Praat phonetic analysis program (version 5.1.43; Boersma & Weenink 2007) to measure the pitch of each utterance (male pitch range 181–207 Hz, mean = 195 Hz; female 91–116 Hz, mean = 107 Hz). Following previous studies [7], [8], [9], we used the Pitch Synchronous Overlap Add Method (PSOLA) algorithm [11] in Praat to convert each utterance into a pair of utterances, one higher and one lower in pitch than the original. The manipulated utterances differed from the original by +/−.5 equivalent rectangular bandwidths (ERB), comparable to a perceived shift of +/−20 Hz. Manipulation by ERB accounts for the nonlinear relationship between absolute and perceived pitch, producing a constant perceivable gap between the higher- and lower-pitched utterances regardless of the fundamental frequency of the original [12]. Our pitch manipulations were perceivable; in pilot work male and female listeners were able to correctly identify the higher-pitched voice of each pair of male and female manipulated voices (*p*<.01 in all cases).

### Procedure

In one experiment, 35 men (mean age = 20.6 years) and 36 women (mean age = 20.4 years) listened to ten pairs of male voices and ten pairs of female voices through headphones connected to a computer. Each pair of voices consisted of the lower-pitched and higher-pitched versions of the same speaker. The pairs of voices were grouped by the sex of the speaker, whereby subjects first listened to the pairs of voices from one sex, and then the pairs from the other sex. We counterbalanced among listeners whether the male or female voice pairs were heard first, and whether the higher- or lower-pitched voice from each pair was heard first. We also randomized the order of the voice pairs within each set. After listening to each pair of voices, subjects responded to the question, “If they were running against each other in a School Board election, which voice would you vote for?” Subjects marked their responses to this “forced choice” question on a paper questionnaire.

In a separate experiment the same procedures were used to assess male (N = 39, mean age = 19.8 years) and female (N = 36, mean age = 20.7 years) subjects’ preferences for President of the PTO. Before voting, subjects read a vignette describing the position: “A Parent Teacher Organization (PTO) is an organization that consists of parents of students, school teachers and school staff. The goals of PTOs include recruiting parents to volunteer in the school, encouraging teachers and students, promoting community involvement, and protecting the welfare of students and families. In some schools, this organization is called a Parent-Teacher Association (PTA) or Parent-Teacher-Student Association (PTSA).” After reading the vignette, in response to listening to each pair of voices subjects responded to the question, “If they were running against each other in an election for PTO President, which voice would you vote for?”.

Prior approval to conduct all elements of the two experiments documented in this paper was granted by the University of Miami (Coral Gables, Florida, USA) Human Subjects Research Office (HSRO) on February 3, 2012 (Protocol ID 20120089). Prior to participating, subjects were presented with a consent form, stating the purpose of the study, procedures to be undertaken, risks and benefits associated with participating, and contact information for the Principle Investigator (CAK) and the University of Miami HSRO. The consent form also explained the subject's right to withdraw from the study at any time, and that participation was confidential (identifying information, such as names or addresses, was not collected). After reading the consent form, subjects were asked whether they understood their rights as research subjects. To document oral consent, subjects were asked to explicitly state their willingness to participate to the research staff administering the experiment. The University of Miami HSRO specifically approved oral consent in the aforementioned human subjects protocol to help maintain the confidentiality of study participants.

### Method of Analysis

The listener is the unit of analysis. Each of the ten forced choices was coded 0 if the listener selected the lower-pitched voice, and 1 if the higher-pitched voice was selected. The average of the ten forced choices yielded a single summary preference ratio for each listener ranging from 0 to 1, whereby higher values indicate a stronger preference for the higher-pitched voices, and lower values indicate a stronger preference for the lower-pitched voices. Using the R statistical computing program (version 2.12.2), two-tailed one-sample t-tests were conducted to compare each sample’s mean preference ratio to.50 (i.e. no preference for higher- or lower-pitched voices).

## Results

### Men’s Preferences

**Figure 1:**
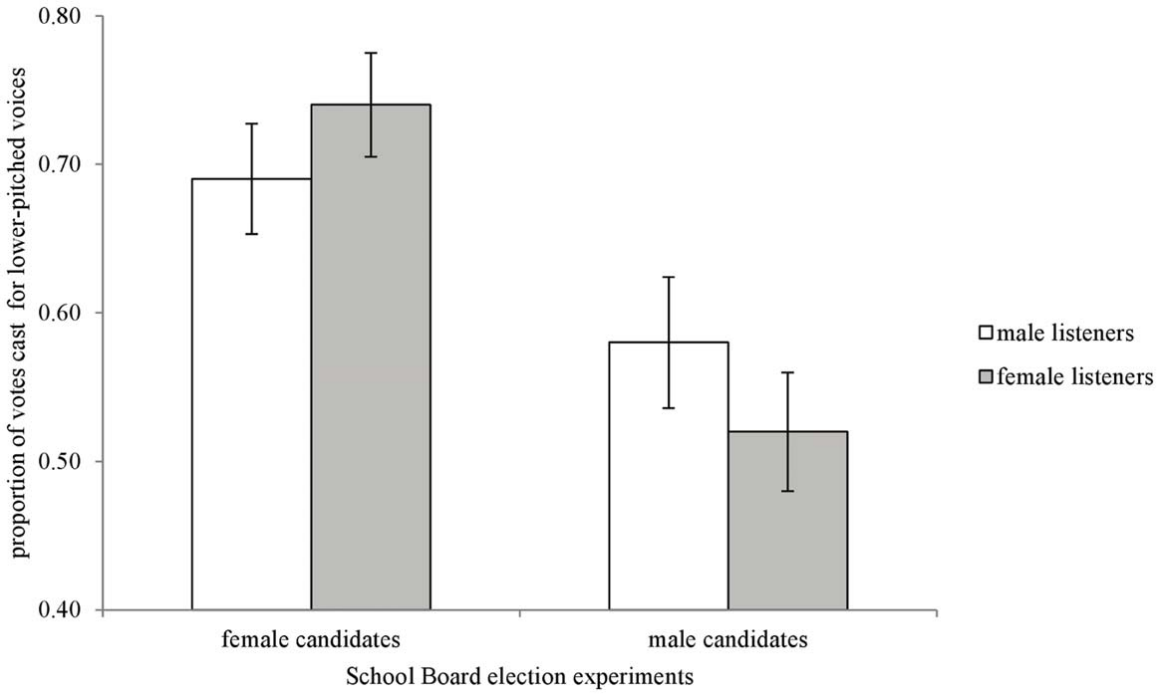
School Board election experiment results. Proportion of votes (+/−SE) cast for the lower-pitched version of male and female voices. A value of.50 represents no discernible preference for either higher- or lower-pitched voices.

**Figure 2:**
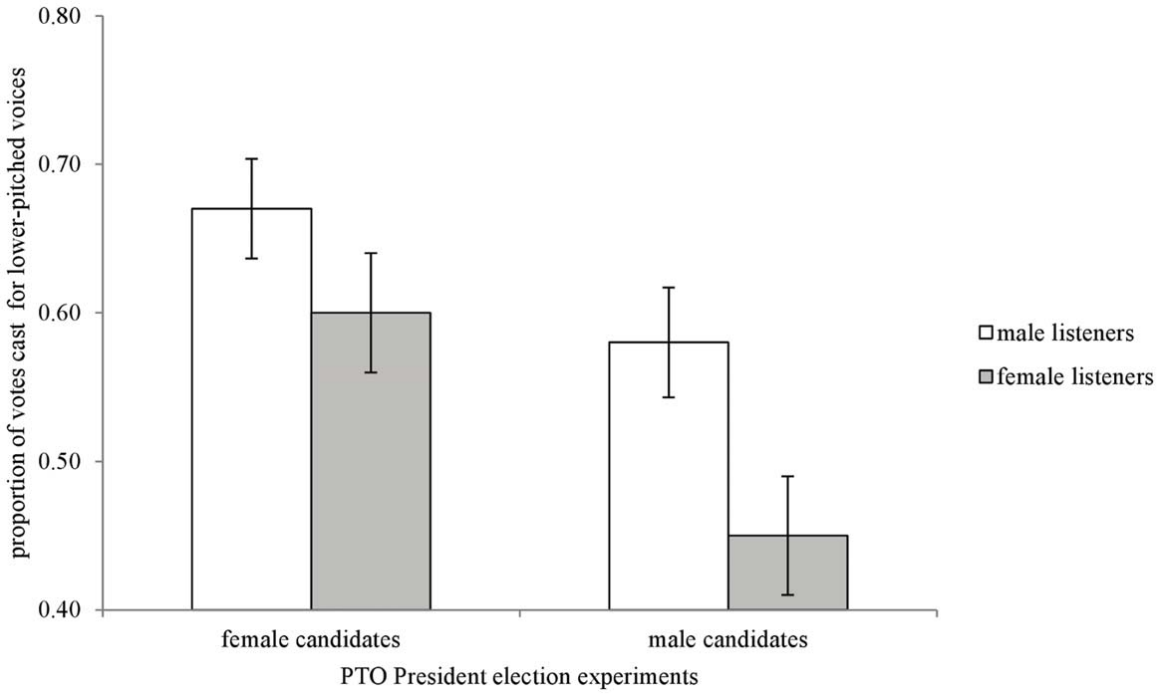
Parent Teacher Association (PTO) President election experiment results. Proportion of votes (+/−SE) cast for the lower-pitched version of male and female voices. A value of.50 represents no discernible preference for either higher- or lower-pitched voices.

As seen in Figure 1, male listeners preferred to vote for male and female candidates with lower-pitched voices running for School Board (male candidates: t_34_ = −3.08, p<.01; female candidates: t_34_ = −5.03, p<.01). Figure 2 shows that male listeners also preferred male and female candidates with lower-pitched voices running for PTO President (male candidates: t_38_ = −2.09, p = .04; female candidates: t_38_ = −5.19, p<.01).

### Women’s Preferences

As in men, Figures 1 and 2 show that female listeners preferred to vote for female candidates with lower-pitched voices running for School Board (t_35_ = −6.73, p<.01) and PTO President (t_35_ = −2.44, p = .02). When asked to judge men’s voices, Figures 1 and 2 show that female listeners did not discriminate between the higher- and lower- pitched voices in both hypothetical elections (School Board: t_35_ = −0.49, p<.62; PTO President (t_35_ = 1.20, p = .24).

## Discussion

The study of human voice pitch perception has expanded into the domain of leadership selection [7], [8], yet the influence of voice pitch on different types of leadership positions had not been addressed. We examined whether the general preference of men and women for male and female leaders with masculine voices shifts to favor men and women with feminine voices in the case of feminine leadership roles (i.e. positions typically occupied by women, or that are congruent with the stereotype of women as caretakers).While feminine qualities are generally perceived to be desirable in individuals holding feminine leadership roles [10], we find that this is not the case for voice pitch. Importantly, despite work showing that listener response to voice pitch can be influenced by social context [9], our results suggest that the influence of voice pitch on perceptions of leadership capacity can be consistent across different types of leadership roles.

Why are men and women with masculinized voices preferred as leaders? In the case of women’s voices, this bias could be a consequence of lower-pitched female voices being perceived as more competent, stronger, and more trustworthy [7]. That is, these traits are perceived as positive in the context of leadership and could be the mechanism that leads us to prefer female leaders with lower voices. Additionally, the pitch of the female voice declines over the lifespan [2], [13]. Consequently, selection of female leaders with lower-pitched voices can result in the selection of women who are older, and perhaps more experienced at leading others. Stated differently, men and women may be biased to select older women as leaders, regardless of the type of position in question. In the case of men’s voices, men with lower-pitched voices are larger, stronger, and more aggressive. Again these traits are perceived as positive in the context of leadership, leading us to prefer male leaders with lower voices.

In contrast to previous studies [7], [8], why do we find that women did not discriminate men’s voices when voting? One explanation for this incongruence is that previous studies used general interrogatives when asking listeners to discriminate between higher- and lower-pitched voices, whereas here we placed the voting task within the specific context of feminine leadership roles. In this specific case, then, the preference of women for male leaders with lower-pitched voices can be moderated. That is, our findings hint at the notion that while men have a consistent preference for masculinized leaders, women may desire men with more feminine qualities in feminine leadership positions. To test this proposition, future studies could ask subjects their preferences for leaders in roles that are more feminized than our examples of PTO President and member of the School Board, such as daycare providers, and other positions that comport with the stereotype of women as caretakers of families and children [10].

Overall, however, our current results are in line with previous studies demonstrating that lower voice pitch is generally preferred in leaders. More specifically, our study is the first to demonstrate a consistent influence of voice pitch across different leadership positions: in general, women and men with lower-pitched voices are preferred by voters in hypothetical election scenarios, whether the elected office is not specified [7], [8] or specified as being commonly held by women (present study). However, ours and other studies are situated within the context of elected offices. What remains to be seen is whether pitch influences the perception of other types of authority figures, such as caregivers and educators. More broadly, additional work is needed to better understand whether, and how, voice pitch relates to individual characteristics that are predictive of leadership capacity.
